# Dynamic capabilities of maritime infrastructure: conceptual design of merchant vessels with usability in crisis

**DOI:** 10.1007/s00773-023-00932-x

**Published:** 2023-03-20

**Authors:** Satoshi Hirayama, Yasuo Ichinose, Shinnosuke Wanaka, Bryan Moser, Kazuo Hiekata

**Affiliations:** 1 Nippon Kaiji Kyokai, Tokyo, Japan; 2 National Maritime Research Institute, National Institute of Maritime, Port and Aviation Technology, Tokyo, Japan; 3 System Design and Management, Massachusetts Institute of Technology, Cambridge, USA; 4 Graduate School of Frontier Sciences, The University of Tokyo, Chiba, Japan

**Keywords:** Crisis recovery, Dynamic capabilities of infrastructure, Hospital ship, Systems architecture

## Abstract

Today’s social infrastructure, e.g., transportation, medical services, energy supply and distribution, may become temporarily unable to provide functions due to the damage to buildings or excessive congestion resulting from threats, such as natural disasters, rising sea levels, pandemics. Maritime-based responses, typified by hospital ships, are drawing attention as a method to mitigate these effects. However, while designing emergency infrastructure, it is necessary to consider not only the value of these systems in emergencies but also during normal times. This study adopts the systems approach, a set of methods to conduct decision-making when complex stakeholders’ relationships are involved. We focus on medical functions and propose a conceptual design for a flexible hospital ship with dynamic capability during emergencies as well as normal times. Specifically, we examine the optimal combination of ship type, size, navigation range during normal times, operations during emergencies, and contract approaches. Quantitative evaluation of utility during emergencies and economic efficiency are considered in tradeoff. In addition to the conventional cost-based study, we examined benefit–cost through ship sharing, in which ships are leased to the private sector as merchant vessels during normal times to generate revenue.

## Introduction

Global society is threatened by natural disasters, such as earthquakes, tsunamis, hurricanes, rising sea levels, and pandemics. Social infrastructure may become temporarily unable to provide functions to meet societal demand due to these crises. Driven by dynamic interplay of technical, social, and economic forces, crises, large or small, force people to move, resulting in gaps between people and infrastructure, reducing the availability of infrastructure not only in the disaster-stricken area but also in destinations where people relocate. Following the earthquake in Haiti in 2010, a study observed the movement of the population using mobile phone location data [1]. The research showed a 23% outflow of the population from the capital city, the epicenter of the earthquake, over 19 days after the earthquake. In addition, in the 2011 earthquake that struck Fukushima, a study studied the movement of the population in the surrounding areas before and after the earthquake based on moving-in and moving-out data [2]. Unsurprisingly, the study confirmed that the coastal areas that were severely damaged by the ensuing tsunami showed a strong tendency of excess relocation after the earthquake. However, inland areas exhibited an increase in excessive new residents, moving-in to municipalities. Thus altogether a sharp increase in movement from coastal areas to inland areas.

Notably, this would not pose a challenge when the infrastructure capacity at the destinations of this population influx would be sufficient to accommodate the additional population—if per-capita infrastructure availability can be adequately maintained. However, depending on the receiving country or region and the volume of population movement, such capacity cannot be ensured, and one of the best examples is refugee crises [3].

**Fig. 1:**
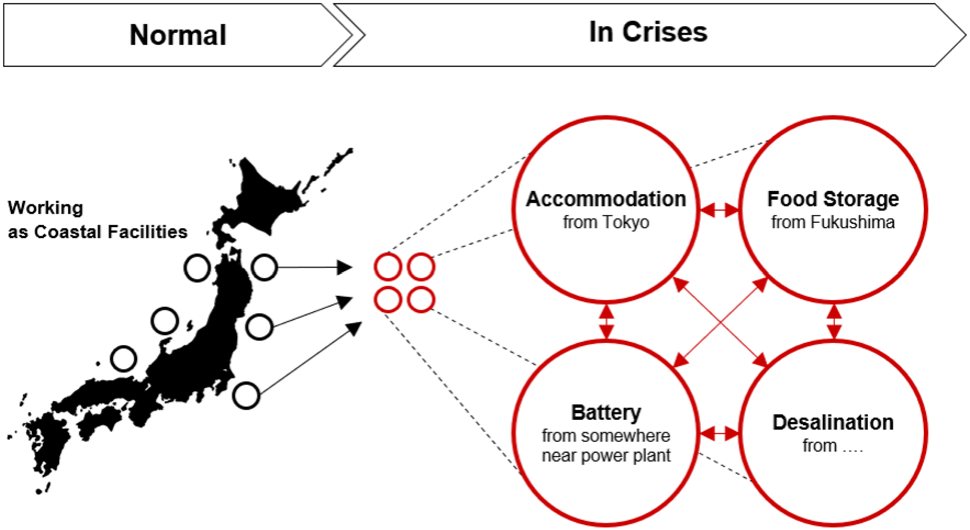
Emergency scenario

One possible means to mitigate the effects of degraded infrastructure function due to crises is a maritime approach that leverages ocean mobility. After the crisis, onshore access to infrastructure may be impeded due to failing infrastructure and excessive congestion caused by population movement, for example damaged roads. Thus, a maritime approach, which supports access to infrastructure by supplementing functions from the sea, can be considered a most promising strategy. For achieving such flexibility and maritime response, we were inspired by an on-demand maritime approach of “sharing the surplus.”(Fig. 1).

There have been studies on creating floating cities and converting coastal areas into maritime communities [4–6] as countermeasures against natural disasters such as tsunamis and rising sea levels due to global warming. This maritime platform infrastructure is engaged in the local economies of coastal areas during normal times. Subsequently, when a crisis occurs, the surplus infrastructure modules, such as backup modules, are assembled around the disaster area to form a single system, providing infrastructure functions that are scarce on land.

Hospital ships are known as a representative method used in the maritime approach [7]. For example, Abdillah et al. [8] proposes a hospital ship design to provide medical services to many islands where medical services are lacking in Indonesia. Amiadji et al. [9] designed catamaran ship propulsion system for the hospital ship. *Peak Ark* is a Chinese naval ship which provides humanitarian medical services for some countries in Africa and Asia [10]. Some cases of transformation of a merchant vessel to a hospital were reported. Cremonesi et al. [11] reported a case of transformation from a ferry ship into a hospital ship for the COVID-19 response in Italy.

Among others, a challenge with these emergency-specific vessels is their unprofitability. Thus, they are infeasible to employ during normal times. The assumptions made about crises involve a high degree of uncertainty. Generally, the duration of emergencies is extremely short compared to normal periods of operation. Therefore, the design of emergency measures should also consider their value during non-crisis normal times. Thus, value in normal times should be an issue when designing crisis-response measures. However, we have found no study that has simultaneously assessed value in emergencies and value in normal times when examining a maritime response to crisis.

In this study, we propose a maritime approach with dynamic capabilities [12] to create a new value by repurposing resources used in normal times for emergencies under highly uncertain conditions.

This study aims to develop a conceptual design for a flexible ship that can function as a regular merchant vessel in normal times and as an alternative to a hospital ship during disasters. Specifically, we examine optimal combinations by assessing utility during emergencies and economic efficiency from the combination of five design decisions: (1) ship type, (2) ship size, (3) navigation area during normal times with consideration for the onboard facilities of a merchant ship, (4) contract type, and (5) operations during emergencies. In addition to a conventional cost-based study, we conduct a benefit–cost study based on the concept of ship sharing in which merchant ships are leased to the private sector during normal times to generate revenue.

## Literature review

In the US, which already possesses hospital ships, Kelso et al. [13] examined the optimal design of the successor ships to the existing hospital ships, MV Mercy and MV Comfort, which are now becoming superannuated vessels. In addition to caring for the wounded in war, they mention another use for hospital ships—providing humanitarian assistance during disasters at home and abroad. Also, the main mission of the successor hospital ships is use in disasters. Technical requirements that should be incorporated into successor ships include improved stability at anchor to (1) improve onboard medical care, (2) reduced draft to facilitate closer access to the shore, and (3) modular medical spaces to expedite the delivery of medical equipment.

In addition, hospital ships have been studied in Japan [14]. The Japanese government, which does not possess hospital ships at present, summarizes the use of hospital ships and merchant vessels carrying medical modules by Japan. The summary highlights potential large-scale disasters, such as the Nankai Trough earthquake or an earthquake that occurs directly beneath the Tokyo metropolitan area, from the perspectives of (1) cost (construction cost, maintenance, and operation costs), (2) constraints and issues related to adoption, (3) possibilities for use in normal times, and (4) the possibility of using private funds. This study further provides detailed data as the basis for decision-making to promote future discussion. Those data confirm that it would not be realistic to consider a general hospital ship owing to an enormous cost to build and maintain it. Furthermore, it would not be possible to use the ship in normal times as an international emergency support ship for disaster relief in other countries, given the chance it would not be available in the event of a disaster in Japan. Thus, use of existing ships, in which commercial passenger vessels are chartered during emergencies and combined with medical modules, is considered a direction for future research. In fact, Ghonanimy [15] studied in detail how containers provide medical service under the COVID-19 situation at Bahrain, therefore, it is sufficiently possible for merchant ships to provide medical service through containers.

Thus, previous studies of maritime approaches have considered improving existing hospital ships and using existing private vessels. However, no conceptual design has been proposed for a flexible ship that provides functionality during normal times, as well as disasters.

The systems approach [16–18] is a set of methods that identify crucial stakeholders in complex problems by organizing interests with a focus on value, making it possible for the design to reflect decisions made in response to stakeholders' needs. Notably, use of the systems approach facilitates a design that entirely meets the requirements of the aforementioned stakeholders.

This approach provides the following procedures emphasizing what to achieve and in a typical order.

- (1) Stakeholder Value Network (SVN) [19, 20].
  A method for arranging complex stakeholder relationships.
- (2) Object Process Methodology (OPM) [21, 22].
  A method for modeling complex systems from a bird’s eye view and another for constructing systems.
- (3) Morphological Matrix (MM) [23, 24].
  A method for organizing decision-making items.
- (4) Tradespace Analysis (TS) [25]
  A method to identify the Pareto front, which is a set of multi-objective optimal solutions with multiple axes of evaluation.

Conflicts in decision-making explain one reason for the slow progress in considering emergency infrastructure designs. Several decisions must be made to create a conceptual idea of infrastructure that can be used in emergencies and be logically feasible. In particular, the combination of two criteria, value in emergencies and value in normal times, make the relationship of interests complex and mutual. Thus, each stakeholder can produce conflicting and unproductive discussions.

A systems approach can help to address the complexity of this collective decision-making. The approach is effective for decision-making with conflicting interests as it enables shared quantitative decision-making, comprehensive visualization of discussion, and reflection of the results of unintended potential decision-making effects. Hence, its role is essential to investigating the design of emergency infrastructure.

This paper presents a conceptual design of a flexible ship that offers functionality both during normal times and disasters. This is proposed using a method that offers an examination using the systems approach. Finally, we derive several concepts to contribute to the future examination of the maritime approach.

## Systems analysis and simulation model

### SVN for the performance metric (PM)

**Fig. 2:**
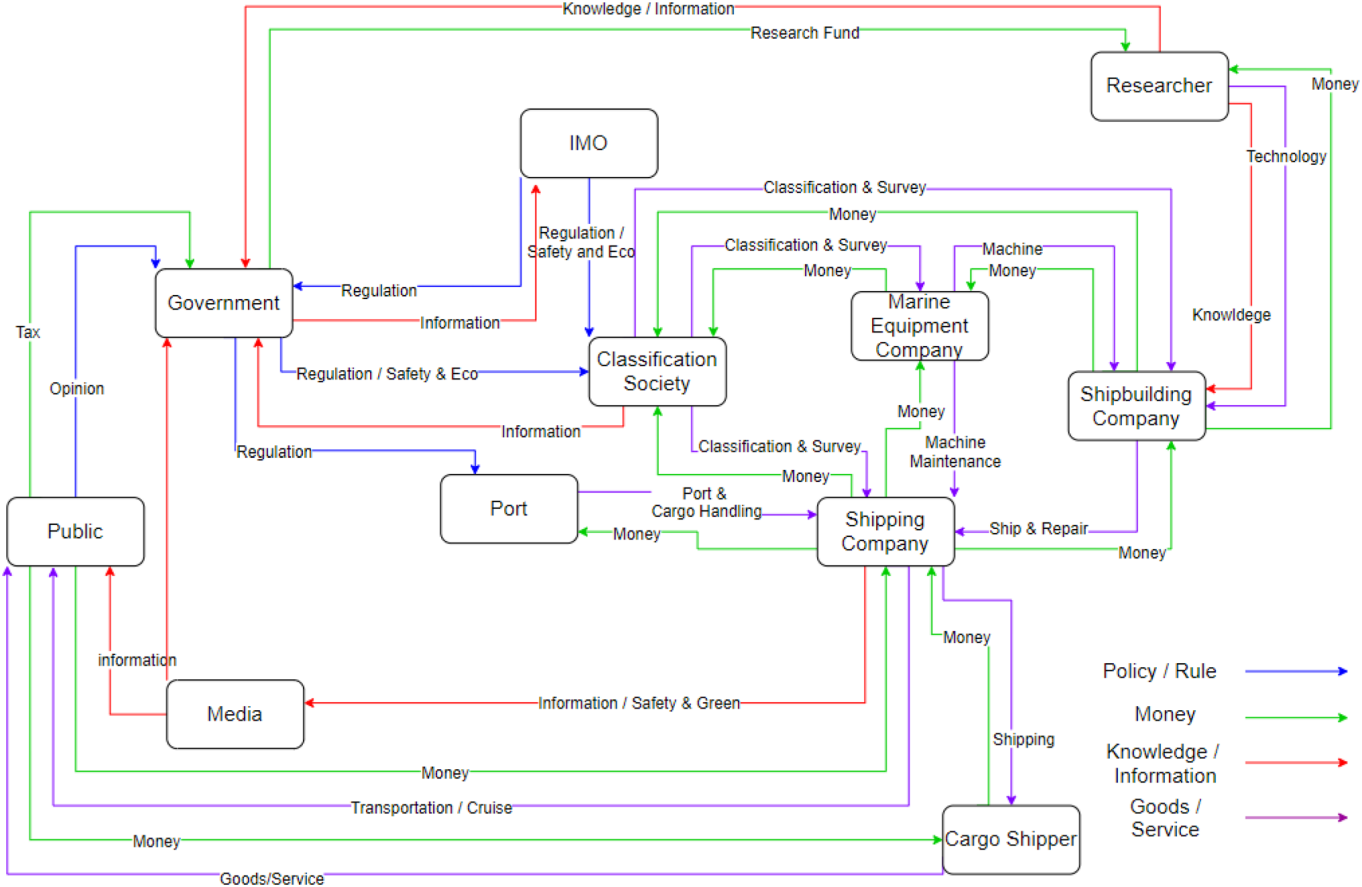
SVN surrounding merchant vessel, adopted from [26]

Hiekata et al. [26] modeled the interests surrounding merchant ships using SVN (Fig. 2). In SVN, stakeholders, such as the government, shipping companies, and residents, are linked by lines of value, such as money, information, goods, and services. Merchant ships are often used by shipping companies in the shipping industry and are depicted as creating social value by creating processes, such as carrying cargo for shippers and transporting passengers.

**Fig. 3:**
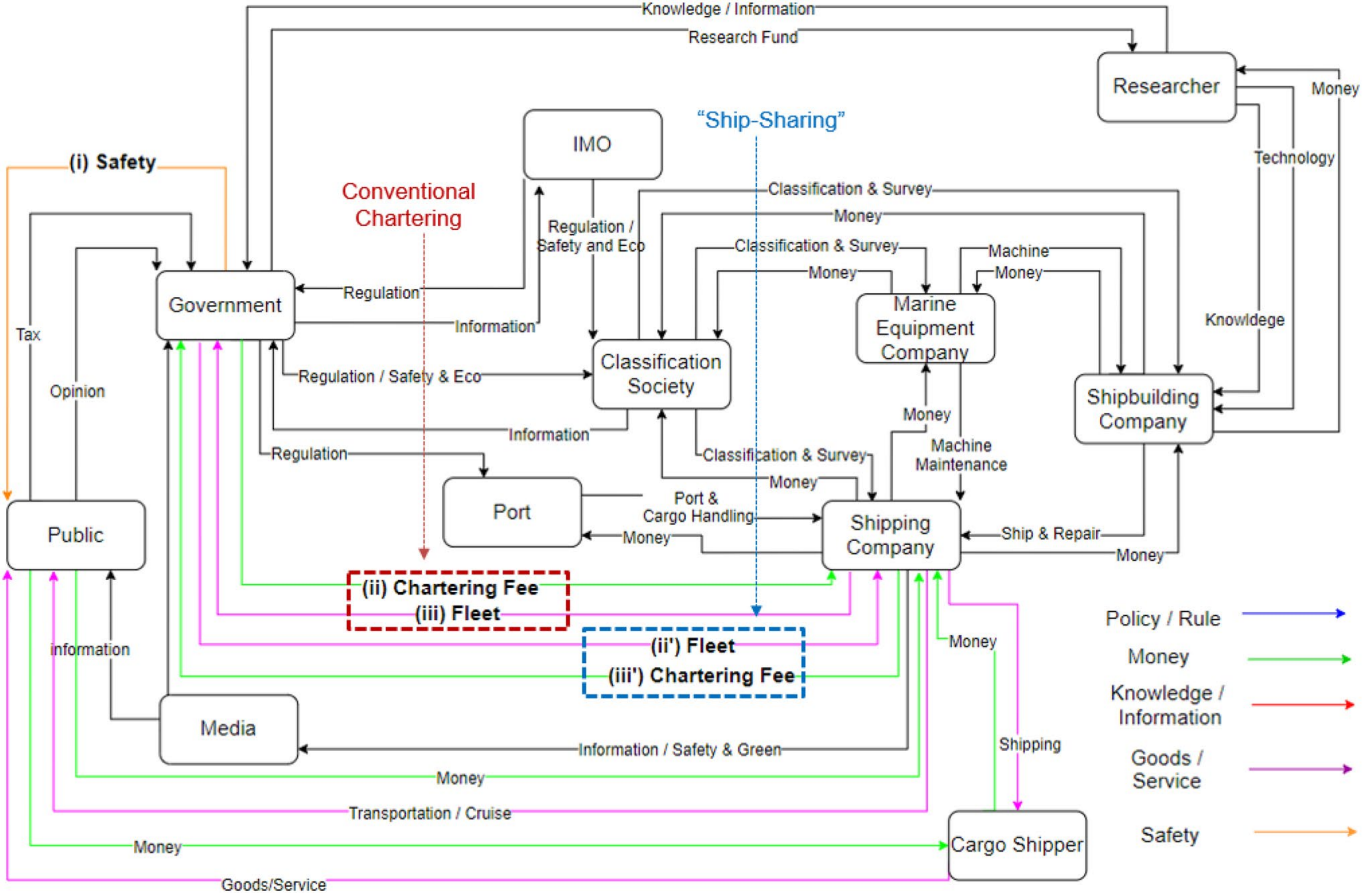
Conventional maritime approach and “ship sharing” concept

Based on the previous studies Fig. 2, we model stakeholders surrounding merchant ships using SVN, which are used as maritime infrastructure in emergency situations as Fig. 3. Figure 3 shows SVN for conventional maritime approach: a hospital ship and, as an alternative, a chartered ship that is temporarily used as a merchant ship, and a new concept called ship-sharing as another operation in emergencies with highlighted in color the value flows with high relevance to use of maritime infrastructure in emergency.

In conventional maritime approach, the stakeholder for an emergency vessel will be the government. The government provides the value of safety (i) to residents using not only hospital ships but also merchant ships on a temporary charter. The government would pay the shipping company a chartering fee (ii), and in return, the shipping company would lease out a merchant vessel (iii). Although it would be necessary to compensate for the difference in facilities from that of a hospital ship, this would create the same value as (i).

In “Ship-Sharing”, however, the government will be the shipowner of this vessel, which will be used as a medical supply vessel during emergencies and leased to shipping companies during normal times. This enables the government to provide safety (i) to the population during emergencies, whereas during normal times, when the government leases ships (ii') to shipping companies, shipping companies can use the government-owned ships to create value for cargo shippers and passengers. Thus, in the opposite direction to conventional chartering, the government earns the chartering fee (iii’).

For the government to establish the ship-sharing scheme, it is vital to incentivize charterers who have different interests from those of the government. For example, if there is a financial incentive, such as lowering the charter rate below the market rate for shipping charter fees, then it will make it easier for charterers to participate. Thus, for the government, ship-sharing will become a new method for considering benefit–cost, in addition to the conventional method for considering hospital ships based on cost alone.

These qualitative assessments using SVN revealed that there are three patterns when considering emergency maritime infrastructure: (1) the government owns general hospital ships, (2) the government charters merchant ships from shipping companies during emergencies, and (3) the government owns the ships and uses them during emergencies while leasing them to shipping companies during normal times. Moreover, a comparison between (1) and (3) showed that ship-sharing contract formats, which do not leave ships idle in normal times, or in other words, where the ship is used to create value in normal times too, also seem desirable.

In this regard, PMs should be prioritized when designing the maritime approach, including not only cost and capability during emergencies but also benefit–cost considering for marketability as a charter vessel. Thus, PMs are set as shown in Table 1.

**Table 1:** Performance metrics

ID	Name	Description	Objective
PM1	Crisis capability	IPM1 + IPM2 + IPM3	Maximize
PM2	Cost	IPM4 + IPM5	Minimize
PM3	Benefit—cost	IPM4 + IPM5 – IPM6	Minimize

Table 2 to be referred to for detail of “IPM”

### Object process methodology for the architectural decision and intermediate variable

To calculate PMs set in Table 1, we derive intermediate performance metrics (IPM), which constitute PMs, and define architectural decisions (AD) to be considered.

**Fig. 4:**
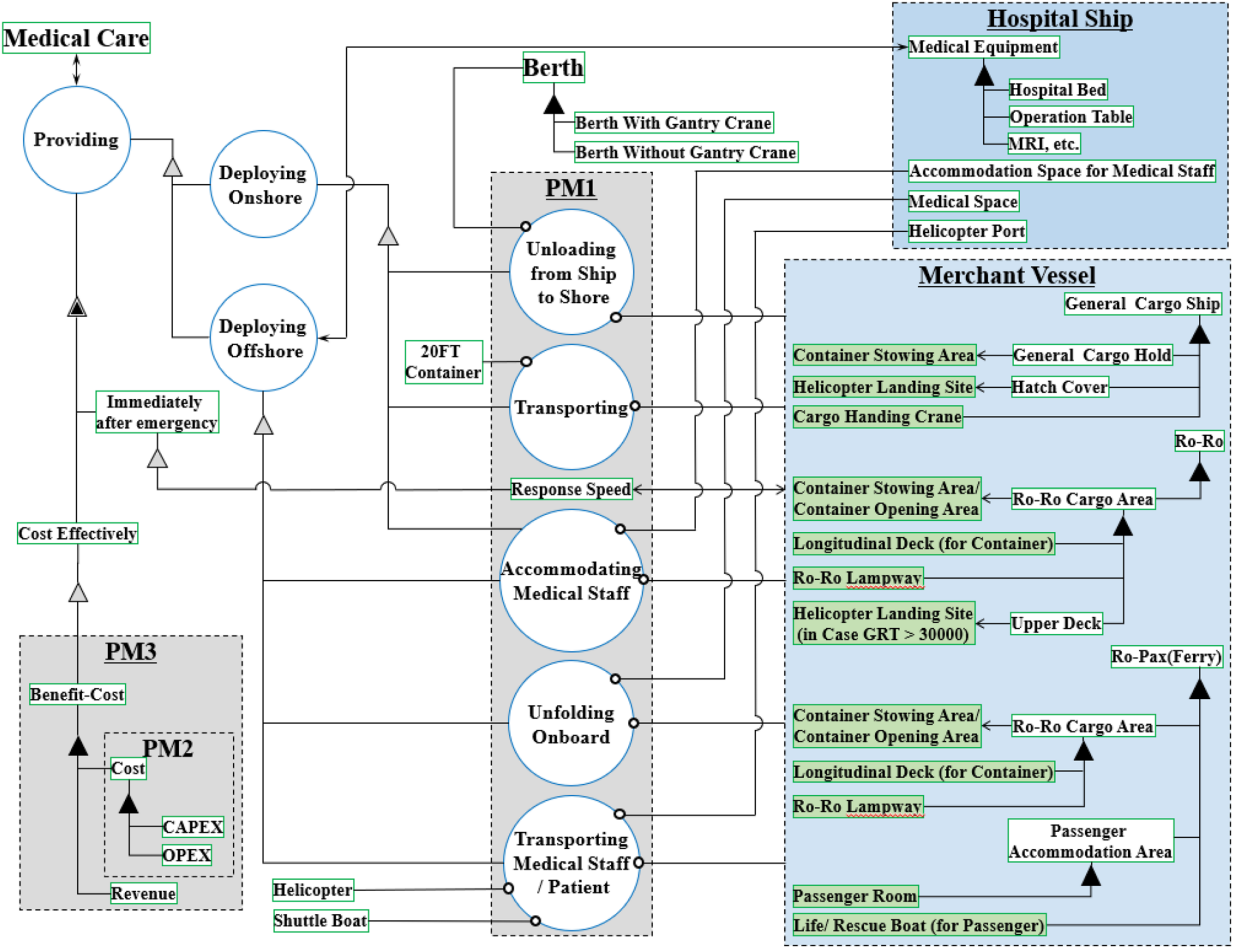
OPM system model of maritime crisis response including dual-use of flexible merchant vessels

We begin by developing a model by object process methodology (OPM) for the maritime system that provides medical functions in emergencies (Fig. 4.).

To design the model as system, we clarified the functions in emergencies referring to the roles anticipated of ships in emergencies on the previous studies [13, 14, 27]. Starting from the upper left in Fig. 4, the general function “Providing Medical Care” is achieved by either the specialized process “Deploying Onshore” or “Deploying Offshore”. Also, they are further specialized in the several processes: “Unloading from Ship to Shore”, “Transporting”, “Accommodating Medical Staff”, “Unfolding Onboard”, and “Transporting Medical Staff/Patient”. These processes are fulfilled by the parts of Hospital Ship which is constructed exclusively for the purpose. Likewise, these processes are also fulfilled by Merchant Vessel that has facilities, highlighted in green, serving as repurposed for emergency use. For example, the process “Accommodating Medical Staff” of PM1 (Crisis Capability) is accomplished by either “Accommodation Space for Medical Staff” of Hospital Ship or “Passenger Room” of Ro-Pax(Ferry). In addition, the attribute “Cost Effectively” of the function “Providing Medical Care” is specialized as PM2 (Cost) consisting of CAPEX and OPEX and PM3 (benefit–cost) of CAPEX, OPEX, and Revenue.

As a result of discussions on Fig. 4 with several experts from the maritime industry, IPMs sensitive to PMs were extracted, as shown in Table 2, which are used in the following simulation of “3.3 Tradespace Model”.

**Table 2:** Intermediate performance metrics (IPM)

ID	Name	Description	Objective
IPM1	Medical capacity	Capacity for deploying 20 ft containers onshore or offshore [TEU]	Maximize
IPM2	Accommodation capacity	Living space for the accommodation of medical personnel [Person]	Maximize
IPM3	Response speed	Speed at which medical care is delivered to disaster areas during the transition from normal times to emergencies	Maximize
IPM4	CAPEX	Cost of building the ship and the fixed cost of medical equipment [m USD]	Minimize
IPM5	OPEX	Vessel maintenance costs, vessel charter costs, medical equipment maintenance costs and leasing costs (per year) [m USD]	Minimize
IPM6	Normal-time revenue	Revenue earned by the government through the leasing of ships to the private sector during normal times (14 years) [m USD]	Maximize

Lastly, various design capabilities are crucial in designing a merchant vessel for normal times; thus, IPM-sensitive design decisions AD are simultaneously extracted using the same method and summarized in Table 3 as a MM.

**Table 3:** Architectural decisions for the maritime crisis response shown in a morphological matrix

No.	Architectural decisions	Option 1	Option 2	Option 3	Option 4	Option 5	Option 6	Option 7	Option 8	Option 9	Option 10	Option 11
AD1	Carrier type	Bulk Carrier	General Cargo Ship	Container Ship	Pure Car Carrier (PCC)	Ro-Ro Ship	Oil Tanker	Chemical Tanker	Gas Carrier	Cruise Ship	Ropax (Ferry)	Hospital Ship
AD2	Size gross tonnage (range)	186918 (200000 - 100000)	87851 (100000 - 50000)	41124 (50000 - 25000)	18418 (25000 - 12500)	11727 (12500 - 6250)	3129 (6250 - 3125)					
AD3	Navigation area in peace-time	International	Domestic									
AD4	Operation type in emergency	Onshore	Offshore									
AD5	Contract type	Government Owning	Government Chartering									

**Fig. 5:**
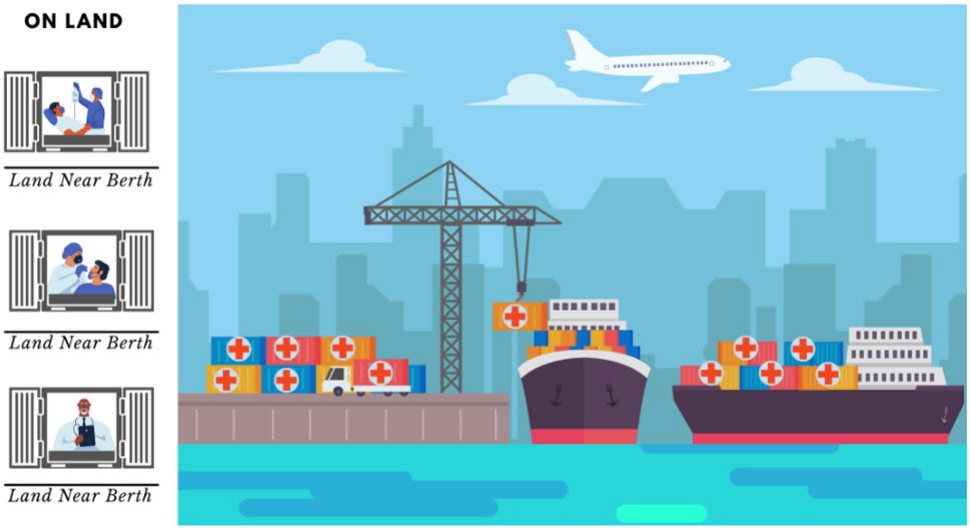
Onshore operation in a crisis

AD4 “Operation Type in Emergency” in Table 3 refers to the type of operations envisioned for the provision of medical care using the ship. In shore-based operations “Onshore”, a vessel transports and unloads a medical container with medical equipment and deploys it in the disaster area (Fig. 5).

One of the characteristics of these operations is that as long as the vessel is capable of carrying containers and has the self-unloading capability to unload containers using onboard equipment, such as onboard cranes and Ro-Ro ramps, various vessels can be used. This has the advantage of expanding the range of vessels available for selection. In contrast, there is the risk that operations may not be feasible where vessels cannot approach land to unload containers due to debris spills, collapsed quays, shallow draft around quays, etc.

**Fig. 6:**
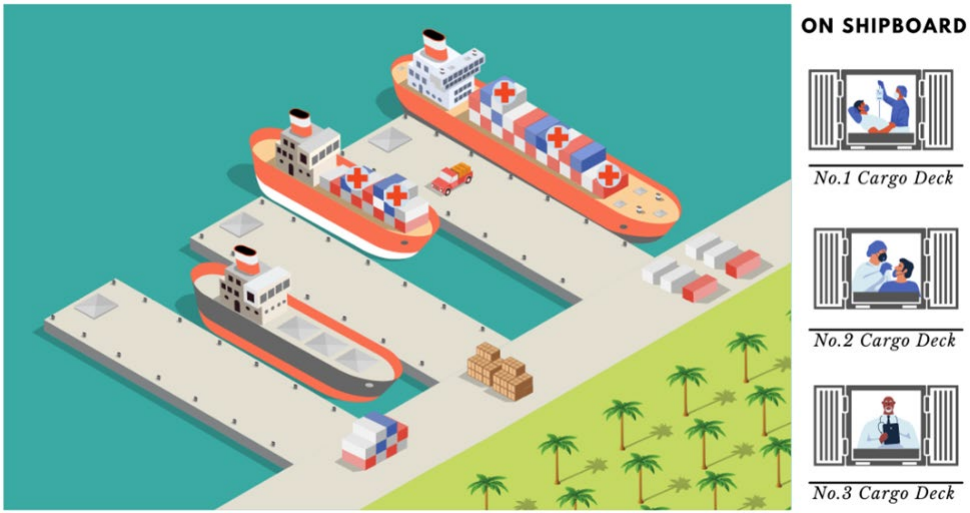
Offshore operation in a crisis

In maritime operations “Offshore”, medical containers are deployed in the cargo holds of ships to provide medical functions onboard (Fig. 6).

Among others, a key feature of offshore operation is utilization of Ro-Ro cargo space in which containers are deployed. Thus, the operation does not necessarily require berthing. As a result, operations are less likely to be impeded by the situation in the disaster area, and operations will be similar to those of a general hospital ship. Conversely, if the ship is unable to dock and provide medical care at sea, it is necessary to rely on other means, such as helicopters or ferry boats, to transport patients between land and ship. In addition, some points must be considered separately, such as the impact of the sway of the ship on medical treatment onboard.

In practice, in addition to medical facilities, one should consider accommodation for medical personnel and the medical personnel themselves. However, this study does not consider securing medical personnel in either onshore or offshore operations and instead emphasizes the provision of facilities.

### Tradespace model

#### Model overview

From the combinations of ADs in Table 3, we derive the respective IPMs in Table 2, and finally, calculate PMs in Table 1 and calculate performance across combinations of ADs.

**Fig. 7:**
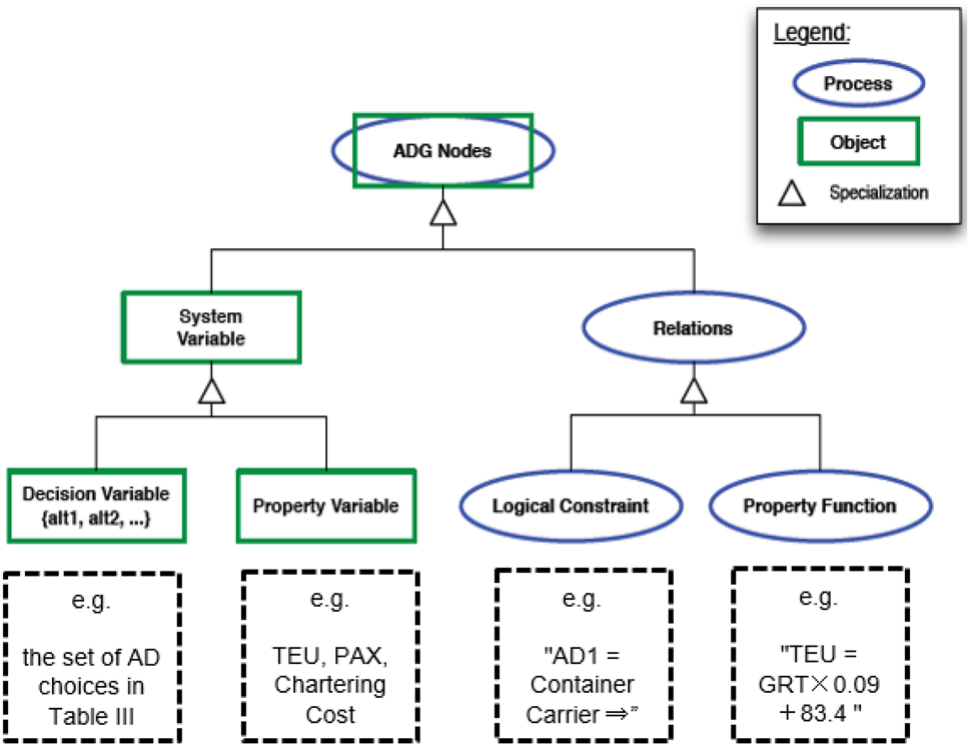
“System variables and relationships” adopted from [28]

In formulating AD, IPM, and PM functions, we adopt an accumulative method according to that proposed by Simmons et al. [28]. Specifically, 4 elements represent the system: decision variables, property variables, logical constraint, and property function (Fig. 7).

*Decision variable* refers to a set of choices that are assigned to variables controlled by the decision maker. For example, the set of AD choices in Table 3.

*Property variable *refers to an area that is discrete or actually assigned value that is a continuous variable. For example, TEU (Twenty-feet Equivalent Unit to indicate Stowage Capacity) and PAX (Passenger Capacity), which vary according to the ship type and size, and ship chartering costs, which vary according to the ship type, size, and shipping market.

*Logical constraint* refers to a conditional constraint that allocates feasible assignments to multiple decision variables. For example, in an TEU calculation—the part in quotation marks in “AD1 = Container Carrier ⇒” TEU = GRT × 0.09 + 83.4.

*Property function* refers to the quantitative role of computing property variables. In an example of the TEU calculation, the part in quotation marks in AD1 = Container Carrier ⇒ “TEU = GRT × 0.09 + 83.4”.

**Fig. 8:**
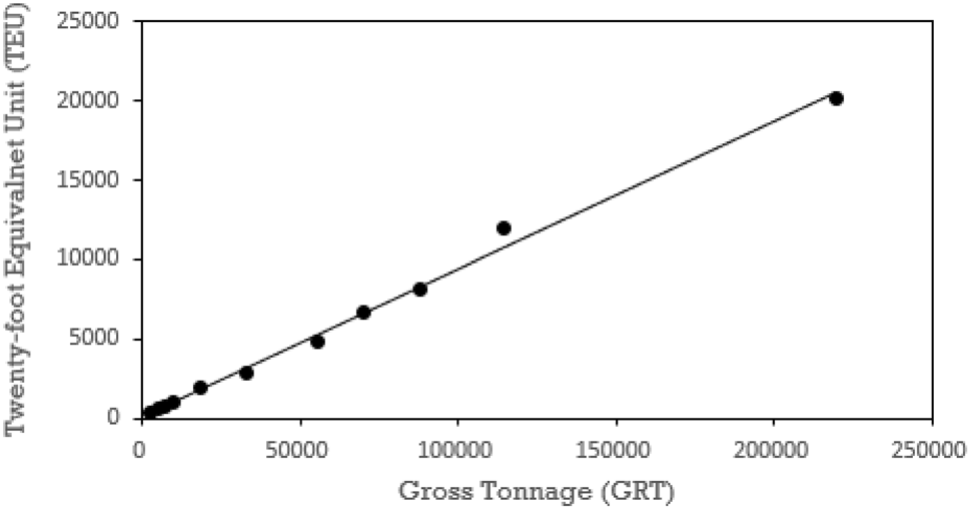
Example of the correlation formula: relationship between GRT and TEU in a container ship

**Fig. 9:**
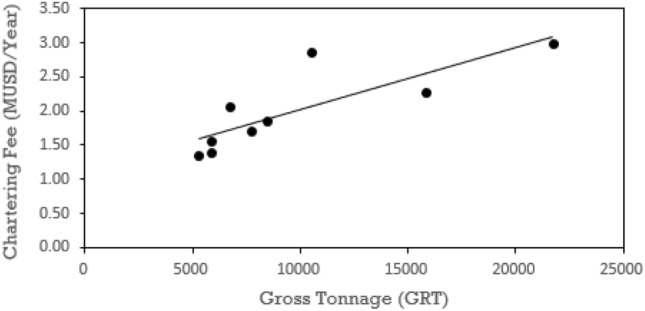
Example of the correlation formula: relationship between GRT and chartering fee in a general cargo ship

In establishing the correlation equation, the characteristics of each type and size of the merchant vessel, the price in the shipping market (construction cost and charter cost), and the characteristics and various costs of hospital ships are examined. Thus, these data were collected from external databases [29, 30] maritime industry interviews, and study reports [13, 14] and prepared using statistical processing. Figures 8 and 9 show examples of this.

By utilizing the Tradespace model, we can quantitatively structure the decision-making process. In detail, the AD selections, which are design options specific to merchant ships, enable the explicit and stable output of PMs. This indicates the emergency capabilities, cost, and revenue calculated via IPM, thereby serving as a decision support tool to determine what kind of merchant vessel design is optimal.

The model is set up assuming a period of 15 years, comprising 1 year of emergency and 14 normal years. The shortage of medical care is assumed to be medical equipment worth 500 hospital beds that is equivalent to one hospital ship. The initial values and constants used in calculations are summarized in Table 3.

#### Crisis capability (PM1)

Crisis capability (PM1) that indicates utility in emergencies was defined as follows.

$${\text{PM}}1_{{\text{Crisis Capability}}} = {\text{IPM}}1_{{\text{Medical Capacity}}} + {\text{IPM}}2_{{\text{Accommodation Capacity}}} + {\text{IPM}}3_{{\text{Response Speed}}} .$$

For the PM1 formula, we referred to the literature on using hospital ships and merchant ships in emergencies, [13, 14, 27] and equalized and summed the respective importance of medical capacity (IPM1), the capacity to provide medical care, the role anticipated of such ships during emergencies, accommodation capacity (IPM2), the facilities to accommodate medical workers, and response speed (IPM3), the speed with which the ship can switch from normal to emergency operations to deliver medical care to the disaster area. Here, IPM1, IPM2, and IPM3 are defined using the logical constraint and property function, respectively:

$${\text{IPM}}1_{{\text{Medical Capacity}}} = \left( {{\text{AD}}4 = {\text{Onshore}} \Rightarrow ({\text{IV}}3_{{\text{container carriage capacity}}} + {\text{IV}}10_{{\text{container discharging ability}}} } \right)/\left( {{\text{IV}}3_{{\left( {{\text{max}}} \right)}} + {\text{IV}}10_{{\left( {{\text{max}}} \right)}} } \right) \times 3) \vee \left( {{\text{AD}}4 = {\text{Offshore}} \Rightarrow ({\text{IV}}4_{{\text{medical equipment unforlding capacity}}} + {\text{IV}}7_{{\text{patient transportation ability}}} } \right)/\left( {{\text{IV}}4_{{\left( {{\text{max}}} \right)}} + {\text{IV}}7_{{\left( {{\text{max}}} \right)}} } \right) \times 3).$$

In the IPM1 equation that expresses the capacity to provide medical care, two types of operations exist: onshore and offshore operations, described in Figs. 5 and 6, respectively.

When AD4 is an onshore operation, it is the sum of container carriage capacity (IV3), which varies according to the input ship type AD1 and size AD2, and container discharging ability (IV10), which is determined by the presence or absence of onboard cranes and Ro-Ro rampways according to the input ship type AD1 and size AD2. More specifically, to decide IV3, we established from statistic data [29, 30] such as Fig. 8 the relation among ship type, ship size, and container carriage capacity. This is exemplified by a case of Bulk Carrier that the TEU = DWT × 0.13; or another case of Container Carrier that the TEU = GRT × 0.09 + 83.4. On the other hand, from the practice of maritime industry, carriage of container on Tanker is not common; therefore the TEU is defined 0. Furthermore, it is formulated such that the patterns of combinations ADs can be compared. For the inclusion of self-unloading capability, a worst-case scenario is assumed in which the gantry crane, a piece of equipment on the receiving wharf, cannot be used due to the effect of a crisis. In addition, determining self-unloading facilities incorporates general merchant vessel design concepts; for example, cargo handling cranes are usually installed on small dry cargo ships.

Similarly, when AD4 is an offshore operation, it is formulated as the sum of medical equipment unfolding capacity (IV4), the capacity to deploy medical containers onboard, which varies according to the input ship type AD1 and size AD2, and patient transportation ability (IV7), which is determined by the availability of helicopter landing site according to the input ship type AD1 and size AD2. To formulate medical container deployment capacity, since it is necessary to open these up on the ship, not in a general TEU, this is formulated at three times volume. When including patient transport capacity, the importance of facilities that can transport patients onboard to land-based hospitals when providing medical care at sea has been highlighted. Also, many hospital ships in an actual operation are designed to be used together with helicopters and feature helipads [13]. Determining the patient transfer facilities incorporates the facility characteristics of typical merchant vessels according to ship type and size. For example, the hatch covers of dry cargo ships and the wide upper decks of large PCC and Ro-Ro ships are actually used as helipads in normal times.

$$\begin{gathered} {\text{IPM}}2_{{\text{Accommodation Capacity}}} = \left( {{\text{AD}}1 = {\text{Hosiptal Ship}} \Rightarrow 3) \vee ({\text{AD}}1 = \neg {\text{Hospital Ship}} \wedge {\text{IV}}2_{{\text{passenger capacity}}} \underline{\underline{ > }} 500 \Rightarrow 3} \right) \hfill \\ \vee ({\text{AD}}1 = \neg {\text{Hospital Ship}} \wedge {\text{IV}}2_{{\text{passenger capacity}}} < 500 \Rightarrow {\text{IV}}2_{{\text{passenger capacity}}} \div 500 \times 3). \hfill \\ \end{gathered}$$

In formulating IPM2, which indicates the capacity of facilities to house medical personnel; notably, the initial condition of the model assumes medical equipment for 500 beds. Since we often assume a situation of 1 member of medical staff per bed [14], we set an upper limit of accommodation facilities for 500 medical staff.

First, when AD1 is a hospital ship with those facilities, it takes a value of 3; when AD1 is not a hospital ship and has passenger capacity (IV2) of 500 or more, it takes a value of 3; and when AD1 is not a hospital ship and has a passenger capacity (IV2) of less than 500, the value is calculated as a proportion of 500. Also, according to the above normalization with 3, IPM1 and IPM3 include multiply 3 for the value weighting to be equal among the IPMs.

$${\text{IPM}}3_{{\text{Response Speed}}} = \left( {{\text{IPM}}1 > 0 \vee {\text{IPM}}2 > 0) \Rightarrow ({\text{IV}}8_{{\text{time to desitination}}} + {\text{IV}}9_{{\text{time for carrier preparation}}} )/\left( {{\text{IV}}3_{{\left( {{\text{max}}} \right)}} + {\text{IV}}10_{{\left( {{\text{max}}} \right)}} } \right) \times 3)} \right).$$

In the response speed (IPM3) equation, which is the speed at which medical care can be delivered to a disaster area after switching from normal to emergency operations, the effect of medical (IPM1) and accommodation functions (IPM2) are added as logical constraints. Subsequently, they are summed with the time to destination (IV8), time determined by the distance to the disaster area, and the time required to procure medical containers, and time for carrier preparation (IV9), which is determined by the time to switch from normal to emergency operations.

In calculating IV8, in addition to whether AD1 is a hospital ship (ship dedicated for emergency or not), it is assumed to be determined by whether AD3, the navigation area in normal times, is international or domestic voyages. In calculating IV9, this was determined by AD5—whether the ship is owned by the government. In determining Response Speed (IPM3), physical differences in ship speed due to differences in the size of the ship and the size of its engines may be considered. Nevertheless, this reflects the results of discussions with the maritime industry about planning matters, such as whether the ship is operating close to land during normal times, and whether the ship is prepared for use in an emergency, as in, whether the ship is a specialized ship, and whether it is owned by the government.

#### Cost (PM2)

Cost (PM2), which represents the cost involved to achieve emergency function, is defined as follows:

$${\mathrm{PM}2}_{\mathrm{Cost}}={\mathrm{IPM}4}_{\mathrm{CAPEX}}+{\mathrm{IPM}5}_{\mathrm{OPEX}}.$$

In the PM2 formula, it is the sum of CAPEX (IPM4), which expresses the fixed cost of emergency infrastructure, and OPEX (IPM5), which represents maintenance costs. IPM4 and IPM5 were respectively defined as follows:

$${\text{IPM}}4_{{{\text{CAPEX}}}} = \left( {{\text{AD}}5 = {\text{Governemnt Owning}} \Rightarrow c1_{{\text{Ship Construction Fee}}} + c4_{{\text{Medical Equipment Fee}}} } \right) \vee \left( {{\text{AD}}5 = {\text{Government Chatering}} \Rightarrow c4_{{\text{Medical Equipment Fee}}} } \right).$$

The IPM4 formula, which expresses the fixed cost of emergency infrastructure, reflects two situations: government ownership and government chartering.

When AD5 is government ownership, it is the sum of the carrier's construction costs (C1), which varies with input ship type AD1 and size AD2, and the fixed cost of medical equipment (C4).

When AD5 is government chartering, then this is taken to be C4 since there is no carrier construction cost. As per Table 4, the value of C4 was set at 76 m USD for the cost of medical equipment for onboard use, where AD1 is a hospital ship, and 140 m USD for containerized medical equipment for other ship types.

**Table 4:** Initial parameters and constants

ID	Description	Value	Unit
–	Period	Emergency 1, Normal 14	Year
–	Medical equipment for 500 hospital beds	290 20 ft.-containers	piece
C1	Hospital ship construction cost	230	m USD
C4	Medical equipment purchasing cost	For hospital ships, 76 In other cases, 140	m USD
C2	Hospital ship annual maintenance costs	5.4	m USD/year
C5	Medical equipment annual maintenance costs	For hospital ships 3, In other cases 1.4	m USD/year

$${\mathrm{IPM}5}_{\mathrm{OPEX}}=\left((\mathrm{AD}1=\mathrm{Hospital \,Ship}\wedge \mathrm{AD}5=\mathrm{Governemnt\, Owning})\Rightarrow {c2}_{\mathrm{Ship \,Maintenance\, Fee}}+{c5}_{\mathrm{Medical \,Equipment \,Maintenance \,Fee}}\right)\vee ((\mathrm{AD}1=\neg \mathrm{Hospital \,Ship}\wedge \mathrm{AD}5=\mathrm{Government \,Owning})\Rightarrow {c5}_{\mathrm{Medical \,Equipment\, Maintenance\, Fee}})\vee (\left(\mathrm{AD}1=\neg \mathrm{Hospital \,Ship}\wedge \mathrm{AD}5=\mathrm{Government\, Chatering}\Rightarrow {c3}_{\mathrm{Shi}{\mathrm{p Chartering \,Fee}}}+{c5}_{\mathrm{Medical\, Equipment \,Maintenance \,Fee}}\right).$$

In the OPEX formula (IPM5), which denotes maintenance costs for emergency infrastructure, there are four possible combinations of (1) whether a ship is the hospital ship and (2) whether it is owned by the government. However, since “AD1 = Hospital ship and AD5 = government Chartering” refers to a privately operated hospital ship chartered by the government, which does not exist, it is excluded, and the following three cases are assumed:

When AD1 is a hospital ship, and AD5 is government-owned, the annual maintenance cost of the ship (C2) and the annual maintenance cost of the medical equipment (C5) are combined.

When AD1 is a merchant vessel, and AD5 is owned by the government, only the annual maintenance cost of the medical equipment (C5) is assumed since it is assumed that the ship is lent to the private sector free of charge in normal times, and the borrower is responsible for the maintenance of the vessel.

When AD1 is a merchant vessel, and AD5 is chartered by the government, the cost of the merchant vessel charter (C3) and the annual maintenance cost of the medical equipment (C5) are totaled.

As in Table 4, the value of C2 is set at 5.4 m USD for hospital ships, whereas C5 is set at 3 m USD for hospital ships, and at 1.4 m USD for the maintenance costs of containerized medical equipment for merchant vessels [14].

#### Benefit—cost (PM3)

Benefit–cost (PM3), which represents the economics of the method used to achieve the emergency function, is defined as follows:

$${\mathrm{PM}3}_{\mathrm{Benefit}-\mathrm{Cost}}={\mathrm{PM}2}_{\mathrm{Cost}}-{\mathrm{IPM}6}_{\mathrm{Peace}-\mathrm{Time\, Profitability}}$$

In the equation for PM3, the cost of emergency infrastructure is PM2 minus IPM6, which is the revenue from lending to the private sector during normal times when the government owns the ship. IPM6 is defined as follows:

$${\mathrm{IPM}6}_{\mathrm{Peace}-\mathrm{Time \,Profitability}}=(\mathrm{AD}1=\neg \mathrm{Hospital \,Ship}\wedge \mathrm{AD}5=\mathrm{Government \,Owning}\Rightarrow {c3}_{\mathrm{Ship \,Chartering \,Fee}}\times 0.5\left({\mathrm{incentive}}_{{\mathrm{for}}_{\mathrm{charterer}}}\right)\times 14\left(\mathrm{year}\right).$$

In the IPM formula, which denotes revenue from lending the vessel to the private sector in normal times when the government owns the vessel, the value of charter rates for ordinary merchant ships is multiplied by the 14 normal years assumed in the conditions of the model.

As an incentive to make this scheme viable, we multiplied the charter rate by a factor of 0.5 to set it to half the charter rate in the general shipping market.

#### Calibration of the model

There were approximately 500 combinations of AD input values. Notably, for example, the following patterns do not exist: AD1 is a PCC, and AD2 is 200000 GRT, implying a substantial PCC; AD1 is a cruise ship, and AD3 is domestic, implying a cruise ship operating domestic routes; AD1 is a hospital ship, and AD5 is chartered by the government, implying a private hospital ship. The results were calculated after the model was modified to exclude these non-existent combinations.

## Simulation results

### Simulation result overview

By constructing the trade-space model up to this point, feasible AD combinations were simulated based on the following four assuming conditions. The ideal case in each result (the top left of the graph) represents the optimal scenario, whereas the dotted line represents the Pareto optimal solution for all simulated concepts.

- 1-1 Assuming provision of medical care at sea (cost–base)
- 1-2 Assuming provision of medical care on land (cost–base)
- 2-1 Assuming provision of medical care at sea (benefit–cost–base)
- 2-2 Assuming provision of medical care on land (benefit–cost–base)

### Simulation result (cost–base)

**Fig. 10:**
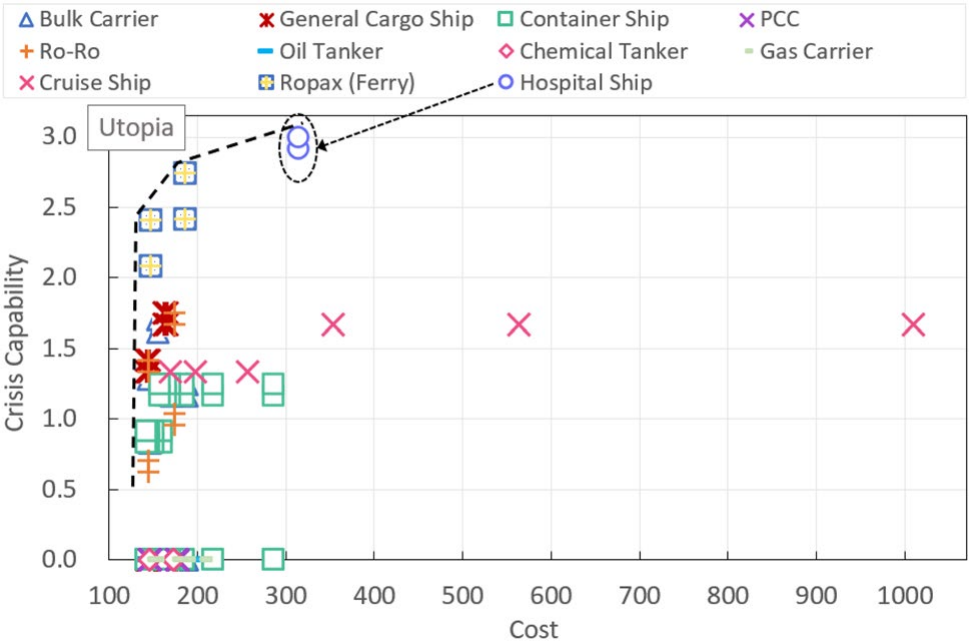
Comparison with the hospital ship (cost-base)

First, the overall cost–base results are shown in Fig. 10.

Based on considerations for general hospital ships, domestic hospital ships, which have high response speeds (IPM3) to the disaster-stricken area by engaging in domestic voyages during normal times, have the highest utility in emergencies. Conversely, merchant ships, chiefly Ropax (Ferry) but also including Ro-Ro ships, General Cargo, and Bulk Carriers, presented close values while keeping costs down.

**Fig. 11.**
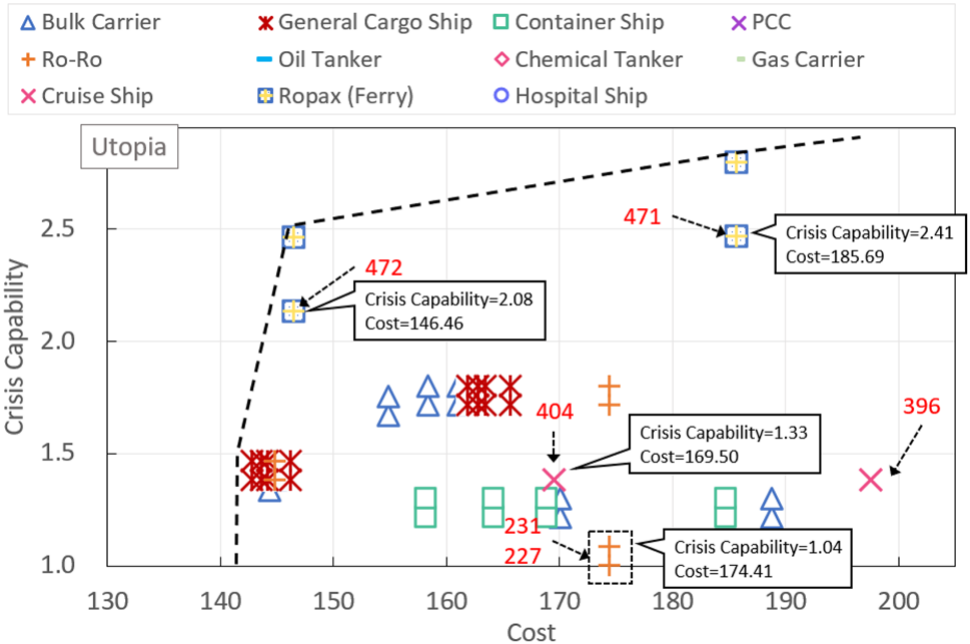
1-1 offshore operation (cost-base)

Next, the detailed results for Case (1-1), which assumes the provision of medical care at sea, are shown as red numbers in Fig. 11, and the discussion can be summarized as follows:

1. 11,727(12,500–6250) GRT Ropax (Ferry): ID472
  Medical containers can be deployed onboard by using the cargo space that is used to load vehicles in normal times. In contrast, accommodation facilities used for passengers in normal times can be used for medical personnel, resulting in a high degree of capability in emergencies. Notably, ID471 has a higher emergency capability in the graph because it is owned by the government and is similar to a general hospital ship in terms of its short carrier preparation time and high response speed. However, in terms of cost, ID472, which has a charter contract where the vessel is chartered by the government, is the Pareto optimal solution. This is because the vessels are chartered; thus, there are no construction costs involved.
2. 41,124(50,000–25,000) GRT Cruise Ship: ID404
  Although it has considerable passenger accommodation facilities, medical containers cannot be deployed onboard. Thus, its emergency capability is not as high as the Ropax (Ferry). However, in this study, it may still be possible to use such a vessel in emergencies if additional considerations are given for carrying medical equipment in accommodation facilities rather than deploying medical containers. ID396 (41124GRT) and ID404 (87851GRT) in Figure 11 take the form of chartered vessels, and the difference in cost is due to the size of vessels.
3. 11,727 (12,500–6250) GTR Ro-Ro ship: ID 231
  Although this ship can deploy medical containers onboard, the upper deck of a Ro-Ro ship of this size is not large enough to arrange a helipad, resulting in a low patient transport capability and consequently a low emergency capability. A large Ro-Ro vessel with a helipad will have higher utility in emergencies. ID231 has lower ship maintenance costs since the government owns the carrier and uses a contract leasing free of charge to the private sector during normal times.
  Since Ro-Ro vessels do not have accommodation facilities for medical staff, it is necessary to additionally consider the transportation of medical personnel by pick-up boats or helicopters, or ship-to-ship operations can be used for accommodation. In that case, since the aforementioned (2) and (3) are specialized ship types for PM2 and PM3, respectively, they may be used in combination. Summing the cost of the two together is approximately equivalent to a hospital ship.

**Fig. 12.**
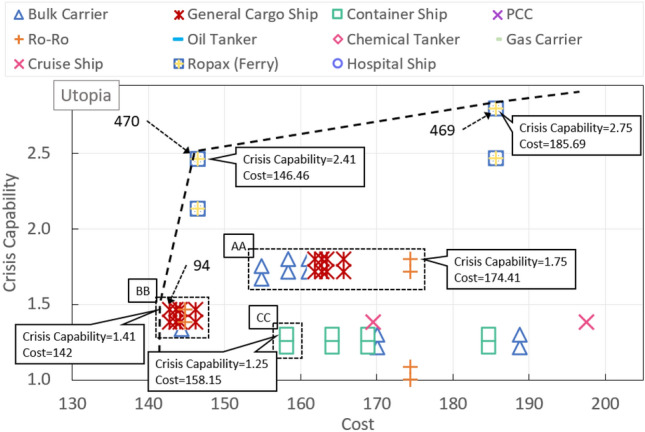
1-2 onshore operation (cost-base)

Subsequently, the detailed results for Case (1-2), which assumes the provision of medical care on land, are shown as black numbers in Fig. 12, and the discussion is summarized below:

1. 11,727(12,500–6250)GRT Ropax (Ferry): ID470
  As with offshore operations, the characteristics of the ship types produces results with high emergency capabilities. The vessel has a cargo space for loading medical containers and a Ro-Ro ramp that enables loading and unloading at the quay, which gives the vessel a high self-unloading capacity, and the vessel also features accommodation facilities for medical personnel. The reason why ID469 in Figure 12 has higher emergency capabilities is that it is owned by the government, which results in shorter carrier preparation times and higher response speeds. Its cost is high, however. The reason why the cost of ID470 is low on the other hand is that its contract type is such that the government charters the ship from the shipowner and so there are no construction costs involved. ID470 is Pareto optimal solution.
2. Cargo ship group AA: Small bulk carriers, general cargo ships, Ro-Ro ships
  In addition to the loading of medical containers, the ships are equipped with onboard cranes, one of the features of small dry cargo ships, and have a high unloading capacity. Since there are no accommodation facilities for medical personnel in this combination, it is necessary to separately consider securing accommodation facilities on land.
3. Cargo ship group BB: small bulk carriers, general cargo ships, Ro-Ro ships
  The difference from (2) above is that these have a charter contract, reducing their cost. However, their emergency capabilities are comparatively low because of the time required for carrier preparation. Among the cargo ships, ID94 is Pareto optimal solution.
4. Cargo ship group CC: 3129(6250–3125) GRT container ship
  In this study, since it is assumed that 290 20-ft medical containers will be transported for the assumed 500 patients, even a small container ship would be sufficient to transport the medical containers. However, since there are no onboard cranes or other equipment on board the vessel to unload containers, the vessel’s own unloading capacity is low and so it is heavily reliant on cranes on the quay. As a result, its emergency capabilities were comparatively low.

### Simulation result (benefit–cost–base)

**Fig. 13:**
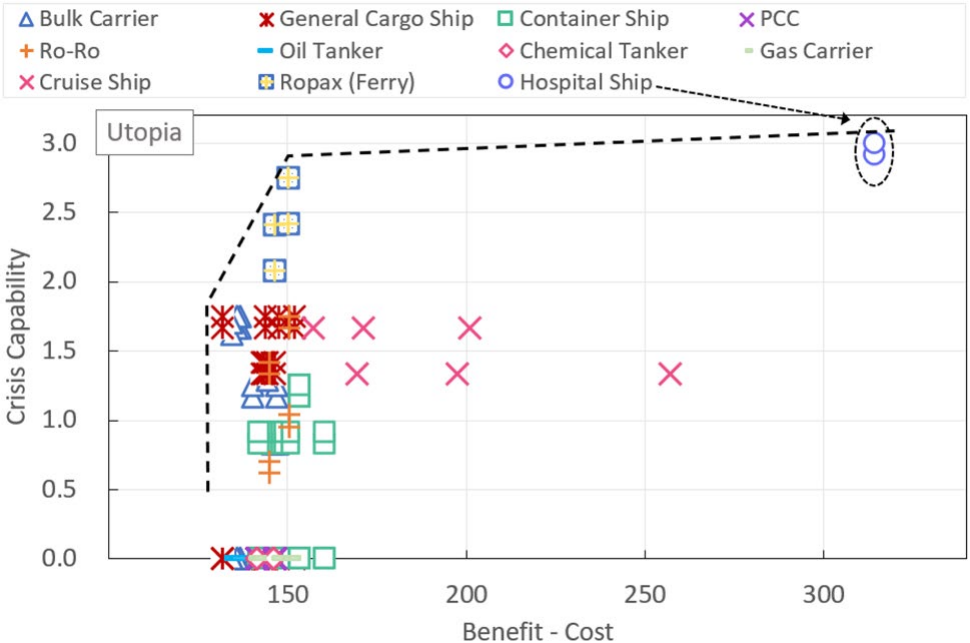
Comparison with the hospital ship (benefit–cost-base)

Next, the benefit–cost-based results are going to discuss. Overall results on a benefit–cost–base are shown in Fig. 13.

The government will own the carrier and it will be used by the government as an alternative of a hospital ship during emergencies but will be chartered out to shipping companies during normal times with incentives (half the typical charter rate), so that the initial expenditure for the carrier will be partially offset by charter revenue. Non-optimal options in cost-focused studies 1-1 and 1-2 became optimal in the benefit-focused studies 2-1 and 2-2.

**Fig. 14:**
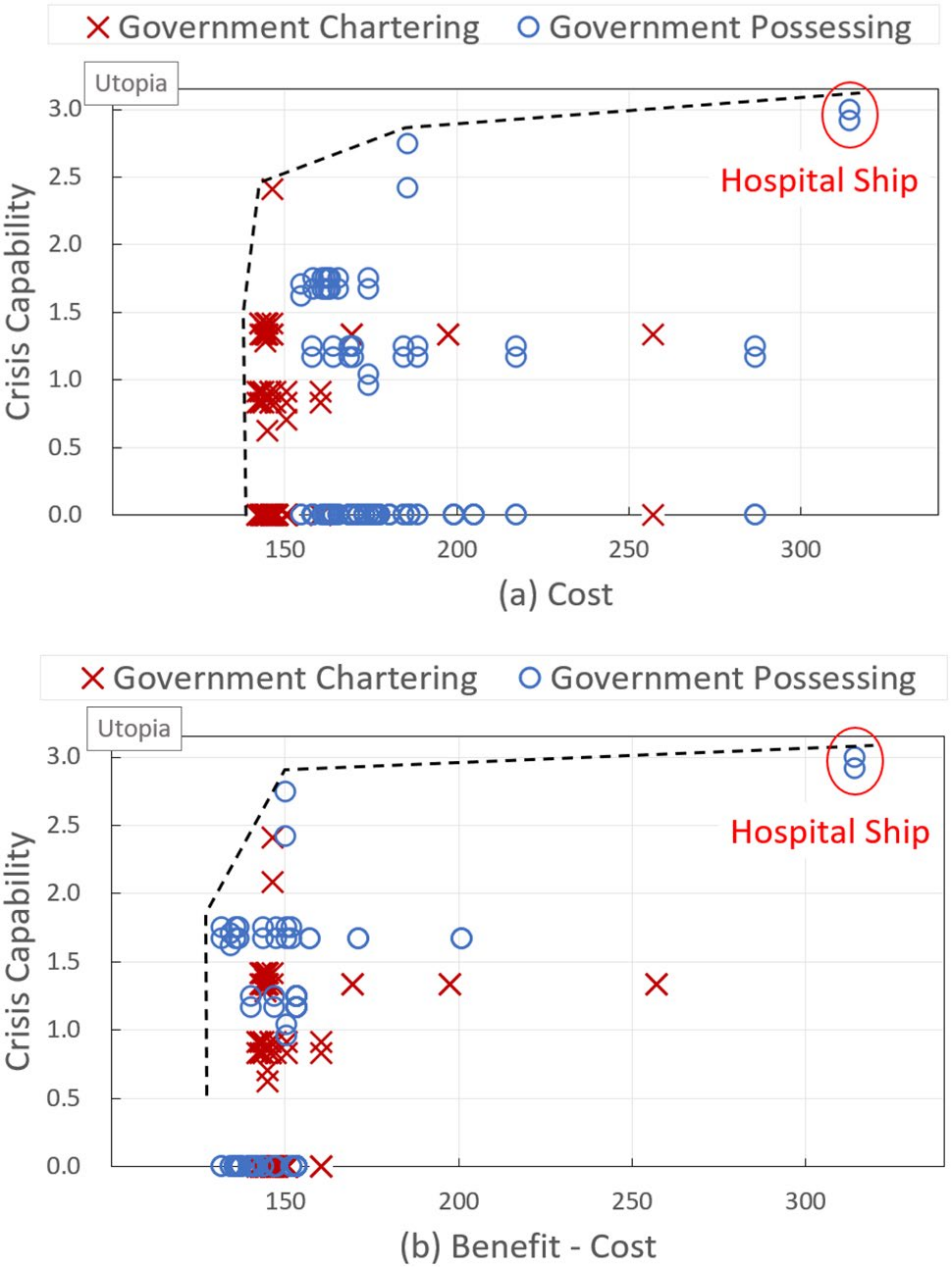
Comparison: cost—base (**a**) vs benefit–cost—base (**b**)

As an overall consideration in Fig. 13, similar to the results of the cost-based study (Fig. 10), merchant ships featuring shipboard facilities with high utility in emergencies followed hospital ships in terms of emergency capabilities. On the other hand, when we look at the cost axis and compare it with Fig. 10, the addition of the revenue component from normal times to offset costs resulted in high cost, non-optimal options on a cost–base approaching the Pareto front, increasing the number of options and moreover, changing the Pareto front (Fig. 14).

Specifically, in cost-based considerations, contracts in which the government chartered vessels (red dot) were predominantly near Pareto—were the best option. In contrast, in the benefit–cost considerations contracts, the government owned vessels (blue dots) were closer to Pareto. This has resulted in more options with higher capabilities in emergencies becoming available.

**Fig. 15:**
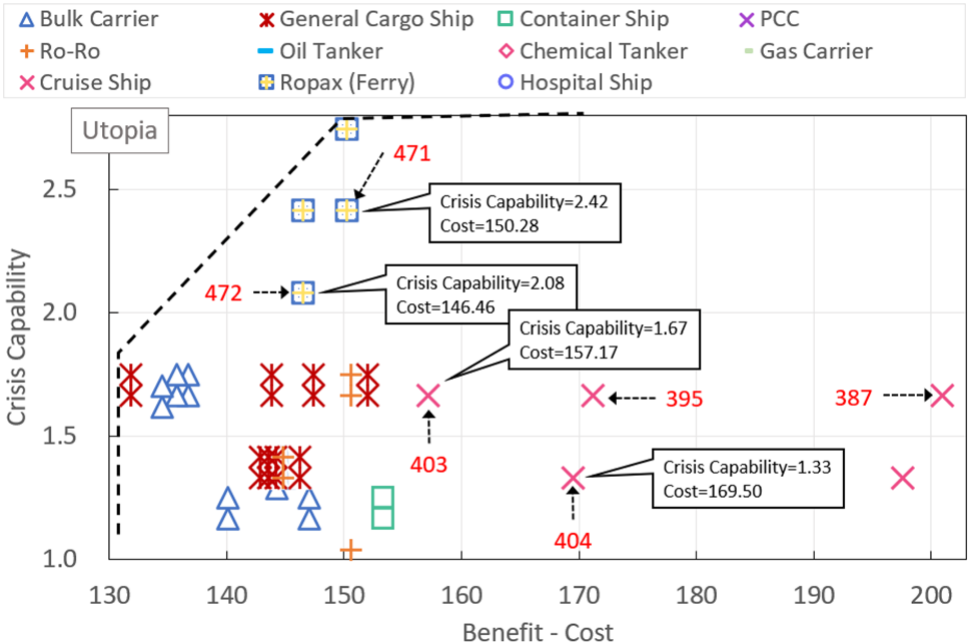
2-1 Offshore operation (benefit–cost–base)

Next, the detailed results for case (2-1), which assumes the provision of medical care at sea, are shown as red numbers in Fig. 15, and the discussion is summarized below:

1. 11,727(12,500–6250) GRT Ropax (Ferry): ID471,472
  In the cost-based study (Fig. 11), ID471 has a higher emergency capability. However, it costs approximately 185 m USD. Thus, ID472 was Pareto optimal. However, ID471 became optimal because their relationship was reduced by considering revenues from contracts in which the government leased the ship to shipping companies in normal times. ID472 is Pareto optimal solution when prioritizing cost.
2. 41,124(50,000–25,000)GRT Passenger ship: ID403
  In the cost-based study (Fig. 11), ID403 has the highest emergency capability among the passenger ships. However, its cost is approximately 353 m USD in size order, which is more expensive than the hospital ship; thus, it is not a candidate. For this reason, ID404 was the optimal choice for the passenger ships, as the contract type was chartered; however, consideration for the profitability of chartering in normal times made it a less expensive option with high emergency capabilities. Of all passenger ships, ID403 became Pareto optimal solution.

**Fig. 16.**
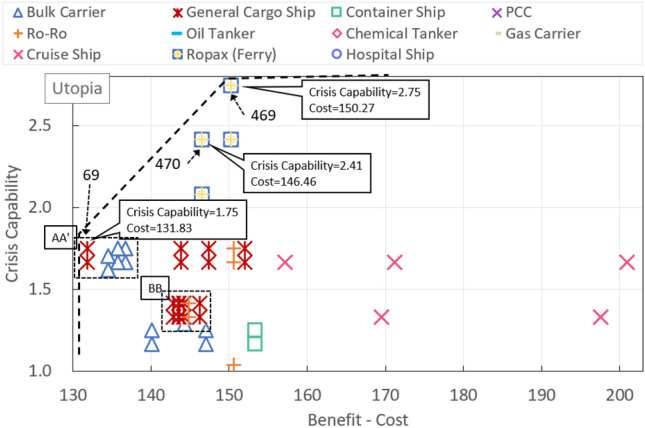
2-2 onshore operation (benefit–cost–base)

Next, the detailed results for case (2-2), which assumes the provision of medical care on land, are shown in Fig. 16, and the discussion is summarized below:

1. 11,727(12,500–6250) GRT Ropax (Ferry): ID469,470
  In the cost-based study (Fig. 12), ID469 has a higher emergency capability; however, it costs approximately 185 m USD; thus, ID470 was Pareto optimal. However, as aforementioned, considering profitability reduced their relationship, and ID469 became optimal. ID470 is optimal when prioritizing cost.
2. Cargo ship group AA': Small bulk carriers, General cargo ships
  In the cost-based study (Fig. 12), the AA group had a higher emergency capability among the cargo ships; however, at the cost of 155-174 m USD.
  Thus, for cargo ships, the BB group was Pareto-optimal when the contract type was a charter. Nevertheless, the AA' group, with its low cost and high emergency capability, demonstrated superior results to the chartered BB group when profitability is considered. Of all cargo ships, ID69 became the Pareto optimal solution.

## Discussion

### Simulation results

In this study, we derived several concepts for flexible ships in two evaluation patterns, one using a conventional cost basis, and the other, a benefit–cost basis in which a ship-sharing concept is introduced. The results are summarized below.

In the *cost* based study, the following concept for offshore operations is attractive: a flexible ship with a multi-story cargo hold with a ceiling height and area enabling medical containers to be deployed on board, cabins for medical personnel to stay on board, helicopter landing site for transporting people between land and ship, and several lifeboats used in normal times for passengers. Specifically, chartering a 10,000 GRT class domestic ferry emerges as an optimal solution. Moreover, the concept of chartering a 40,000 GRT class ocean-going passenger ship and government ownership of a 10,000 GRT class Ro-Ro ship (during normal times, where the ships are leased to the private sector free of charge to reduce ship maintenance costs) was also considered. However, since these ships specialize in medical care provision and accommodation capabilities, respectively, it became necessary to consider the missing capacity separately (e.g., the passenger ship and the Ro-Ro ship could be connected via ship-to-ship operations, with the disadvantage of more complicated operations). As a concept for onshore operations, it is observed that a vessel with a cargo hold capable of carrying medical containers and crane or Ro-Ro ramp capable of unloading containers, would be apt for use as a flexible vessel. Specifically, chartering a 10,000 GRT class domestic ferry became an optimal solution. The chartering of a small domestic general cargo ship was also considered as a candidate; however, it was necessary to consider accommodation facilities for medical personnel separately (e.g., securing accommodation facilities ashore).

In the benefit–cost study, a 10,000 GRT-class domestic ferry, followed by a 40,000 GRT-class ocean-going passenger vessel, were optimal as offshore operations concepts. However, because of the characteristics of the ship types, ship sharing, rather than chartering, changed the set of Pareto optimal solutions. In addition to offsetting the cost of retaining carriers for emergency operations using revenue earned during normal times, this contractual arrangement increases the speed of response to disasters: how the vessel is used during emergencies, the preparation of facilities, and operations and separation from duties during normal times are defined on a contractual basis. Thus, the optimal combination given a benefit–cost basis has a higher utility in emergencies than in the cost-based examination. For the same reason, the concept of public–private sharing of 10,000 GRT-class domestic ferries, followed by small general cargo ships and bulk carriers, was also raised for onshore operations.

### Sensitivity analysis

**Fig. 17:**
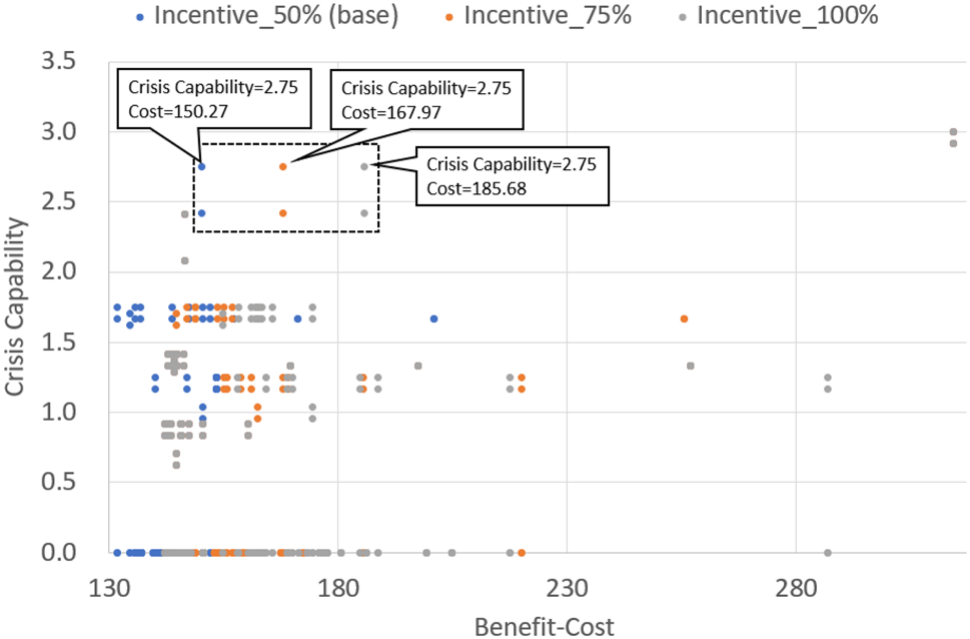
Sensitivity analysis (“incentive_for_charterer”)

**Fig. 18:**
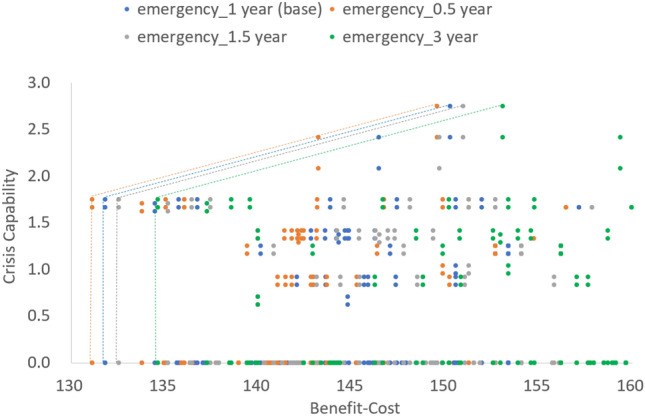
Sensitivity analysis (emergency period)

To discuss the model assumption in depth, we carried out sensitivity analysis for the following parameters:- “incentive_for_charterer” mentioned in the formula “IPM6” in chapter 3.3.4 benefit–cost (PM3) as Fig. 17—Emergency Period in “Table 4. Initial parameters and constants” as Fig. 18.

Initially, in order for private shipping company to easily adopt ship-sharing scheme, the charter rate was decreased in half compared to the general shipping market rate with a factor of 0.5 multiplied. Additionally, we added two cases that increase incentive rate for shipping company: 75% and 100% which means government doesn’t receive profit from shipping company even when leasing the vessel. To easily understand the result tendency, the wavy line rectangle in Fig. 17 shows the more incentive for charterer is prepared, the more cost amount for government becomes without affecting the relative positions of optimal combination of architectural decisions: 11,727(12,500–6250) GRT Ropax (Ferry): ID469. Also, the tendency applies to other parts as shown in the graph.

In addition to the emergency period of 1 year which is the base condition for our simulations, we carried out simulation with 0.5 year, 1.5 year and 3 year as emergency period. As the result, the relative positions of optimal combination patterns remains unchanged as indicated with each pareto-front.

Therefore, we concluded changes in the assumption we set for simulations don’t affect the results.

### Limitation of study

In a future study, the precondition of the simulation should be reconsidered based on each actual case. Crisis has huge uncertainty in terms of risk that, for example, differs among regions. In this study, we set up assuming period as 15 year in total, comprising 1 year of emergency and 14 normal years in terms of the frequency of crisis breaking out. Also, the same applies to the severity, which is assumed in this paper as 500 hospital beds needed. Therefore, this simulation results serves as a case study based on our precondition; and to be accurate in a future study, these assumption values should be adjusted.

Second, the financial calculation including CAPEX, OPEX, and revenue by leasing is roughly defined just to see the relative positions of optimal decision patters in comparison, focusing on developing an early-phase concept. To be on the safe side, we additionally carried out sensitivity analysis in Fig. 17 and Fig. 18; however, it should be mentioned here that the detailed calculation method with better accuracy such as discount rate for the useful life of 15 years is not considered in this paper.

Finally, a further simulation model that carefully considers ship operations would be of value to the study of the dynamic capability of maritime infrastructure. Specifically, the proposed models have a limitation for new ship types built upon existing merchant vessels adding onboard facilities that offer high utility during emergencies. For example, although adding a crane to a conventional small container ship to increase its self-unloading capacity can be estimated, the economic impact of the addition on operations in normal times has not been examined. In addition, especially when it comes to the metrics of Response Speed, the availability of vessel should be discussed in depth. A practical topic is whether the “ship sharing” contract or the carriage contract used in normal times takes priority when the vessel is engaged in the carriage of cargo or passengers when switching from normal operations. Specifically, it is necessary to consider the metrics of "ease of switching from normal operations" according to the type of transportation performed during normal times, such as ease of switching for leisure passenger vessels compared to cargo ships carrying resources.

By refining this model to include such elements in the future, we hope to observe conceptual designs for flexible ships with more significant utility in emergencies.

### Further application of study

In this study, to examine flexible ships characteristics and sizes, we have considered shipbuilding design principles and trends, along with the type of ship contract. Particularly, the novel contribution is introduction of the ship-sharing concept and profitability in non-crisis periods. The proposed method can be applied to design multipurpose infrastructure that modifies value creation by repurposing resources for emergency and normal periods also requiring versatility.

This study is based on a targeted scenario in which medical containers and merchant ships are used as compensatory alternatives for declines in medical function, a social infrastructure due to natural disasters or pandemics. In addition, as mentioned in the introduction, a broader view of social infrastructural capacity should reflect the ripple effect that crises have on the movement of people, albeit on different scales. Therefore, we expect that this study will be used to create means of providing infrastructure functions in response to crises with similar impact structures, such as rising sea levels and refugee issues.

## Conclusion

In this study, we explored and selected a conceptual design for a flexible vessel that functions as a regular merchant ship in 14-year-normal times and as an alternative to a hospital ship in a 1-year-disaster. Specifically, we used the systems approach to evaluate the utility and economic efficiency of a combination of five design decisions for flexible merchant ships: ship type, size, navigation area in normal times, emergency operation type, and contract type. In addition to a conventional cost-based study, we conducted a benefit–cost study, which includes ship-sharing in which merchant ships are leased to the private sector during normal times.

First, the findings of the study are that, compared to owning the general hospital ship whose cost is 314 m USD, the flexible ships could be of economic choices: 146–185 m USD in conventional contracts that government temporarily charters a useful merchant ship from shipping company or that government initially builds and uses it only in emergency while shipping company making good use of it just by paying maintenance fee instead of government in normal periods. In addition, the ship-sharing contract widens the choices for government: 131–150 m USD by incorporating the scheme of leasing the ships to shipping company. On the other hand, for the flexible ships to perform as equivalently as hospital ship which is designed and operated exclusively for emergency use, all the components of maritime crisis response system including not only flexible ships but also berth, medical containers, and transportation means must be well arranged and work in-depth with each other.

Second, the further findings of flexible ship specification follow in detail: on a cost basis during normal times, if the ships have an offshore operation, ships with multi-story cargo holds, and cabins for passengers and ships with large space on their upper deck, or a lifeboat are suitable as the flexible ships. The multi-story cargo holds are utilized to deploy medical containers onboard, and the cabins for passengers become space for medical staff to stay onboard. The large space on the upper deck is required for helicopters’ landing and the lifeboats. Also, the space for helicopters enhances the accessibility of ships under emergency. In the same cost-based studies, the ships which have onshore operations do not require facilities that enable the transfer of people between land and ship. Conversely, it requires a cargo hold capable of carrying medical containers, cranes, and Ro-Ro ramps capable of unloading said containers.

Third, the final findings are that, by adopting the concept of ship-sharing, concepts that were not attractive in the cost-based study due to their high costs became optimal. Specifically, a 10,000 GRT-class domestic ferry and a 40,000 GRT-class international passenger ships shared by government and private sectors were appropriate as flexible ships for the offshore operations concept although additional consideration should be given to the delivery of medical equipment in the latter. In addition, for onshore operations a 10,000 GRT-class domestic ferry and small general cargo ship or bulk carrier are suitable as flexible vessels shared by the government and private sectors although it will be necessary to additionally consider securing accommodation for medical personnel on land for the latter ship type.

## Data Availability

Data sharing is not applicable to this article.
